# Supplementary material for: Differential contributions of static and time-varying functional connectivity to human behavior
Source: Netw Neurosci. 2021 Feb 1;5(1):145–65. doi: 10.1162/netn_a_00172 (PMC7935045; doi:10.1162/netn_a_00172)
Supplement: Supplementary file 1 [file netn-05-145-s001.pdf]

Eichenbaum, A., Pappas, I., Lurie, D., Cohen, J. R. & D'Esposito, M. (2021). Supporting information for "Differential Contributions of Static and Time-Varying Functional Connectivity to Human Behavior." *Network Neuroscience.* 5(1), 145–165. https://doi.org/10.1162/netn_a_00172

Supplementary Materials

Phase-Randomized Surrogate Analysis

Following the approach of Prichard et al. (1994) and Hindriks et al. (2016), we created phase-randomized surrogate timeseries for all available subjects and sessions. As the randomization of phases in a timeseries has no impact on the static nature of the data (i.e., the static correlation matrix produced from the real data and a phase-randomized surrogate set will be identical), these analyses are focused only on time-varying FC.

We first created 250 phase-randomized surrogate datasets as per Hindriks et al. (2016). We then fit the HMM to each of these datasets individually, using the exact same model parameters as used in the main text. We next submitted the HMM-derived time-varying FC metrics (i.e., fractional occupancies and switching rates of each state and metastate) from each surrogate set to a CCA alongside the 8 behavioral factors. Of these 250 surrogate sets, all produced HMM fits that resulted in a 3 metastate profile, however variability existed in the number of states clustered together within each metastate. When submitting these data to the CCA, 1 (0.4%) produced 0 significant modes of covariation, 102 (40.8%) produced 1 significant mode, 141 (56.4%) produced 2 significant modes, and 6 (2.4%) produced 3 significant modes. In order to be consistent with the real data, we isolated the 141 datasets that resulted in 2 modes of covariation and determined how similar these modes were to the modes discovered using the real data.

For these 141 datasets and following the same rationale as in the main text, we performed post-hoc correlations between the CCA fitted data and behavior, as well as between the CCA fitted data and time-varying FC. Specifically, the procedure was as follows:

(1) We correlated the time-varying FC-respective CCA-fitted behavioral data (i.e., V, which is an n x m matrix in which n is the number of subjects and m is the maximal number of possible modes of covariation, which in our case is 8 as there are 8 behavioral factors) with the original behavioral data matrix (an n x r matrix in which r is the number of behavioral measures originally submitted to the factor analysis [r = 31]). This method reveals to degree to which the canonical covariate mode is explained by each behavioral measure. (2) We repeated this procedure for the behavior-respective CCA-fitted time-varying FC data (i.e., U, which is also an n x m matrix), whereby we correlated that data with the original 17 HMM-derived measures; (3) We stored the resultant 31- and 17-element vectors of post-hoc correlations (behavior- and time-varying FC-respective) for the 1st and 2nd mode of covariation from each surrogate set; (4) In order to see whether the surrogate data and real data produced similar results, we correlated those vectors with the post-hoc correlation vectors produced using the real data, and stored all correlation values for the behavior- and time-varying FC-respective sets separately.

We first assessed the similarity between the real data and the surrogate data using the post-hoc correlations derived from the 1st discovered mode. For the behavior-respective post-hoc correlations, we found that 134 (out of 141) surrogate sets explained more than 25% of the variance in the original dataset, with 125 of those explaining more than 50% of the variance. For the time-varying FC-respective post-hoc correlations, we found that 8 (out of 141) surrogate sets explained more than 25% of the variance in the original dataset, with 5 of those explaining more than 50% of the variance.

We next assessed the similarity between the real data and surrogate data using the post-hoc correlations derived from the 2nd discovered mode. For the behavior-respective

post-hoc correlations, we found that 104 (out of 141) surrogate sets explained more than 25% of the variance in the original dataset, with 64 of those explaining more than 50% of the variance. For the time-varying FC-respective post-hoc correlations, we found that 7 (out of 141) surrogate sets explained more than 25% of the variance in the original dataset, with 1 of those explaining more than 50% of the variance.

As a mode of covariation is defined by the fitting of weights to both sides of a dataset (i.e., both behavior and time-varying FC), it is important to consider how null model analyses compare to both sides of the dataset. For the behavior-respective post-hoc correlations between the real data and the phase-randomized surrogate data, the behavioral profile was quite similar to that found using the real data, particularly for the 1st mode. Specifically, 50% (i.e., 125 / 250 for mode 1) and 25.6% (i.e., 64 / 250 for mode 2) of surrogate datasets produced post-hoc correlations that explained more than half the variance of the original dataset. However, both the 1st and 2nd modes of covariation found using the surrogate sets failed to resemble the time-varying FC profile found using the original data, as only 2% (i.e., 5 / 250 for mode 1) and 0.4% (i.e., 1 / 250 for mode 2) of surrogate datasets produced modes that explained more than half of the variance in the original data. These findings prompt the following considerations.

First, all surrogate sets produced a 3-metastate profile when fit to the HMM. This outcome likely aligns with previous HMM analyses (Vidaurre et al., 2017) wherein simulated datasets using autoregressive modeling without phase-randomization produced HMM fits that resulted in a 3-metastate profile. While the overall pattern of metastates was similar, potentially reflecting clusters of subject-specific temporal correlations existing in both

surrogate and real data, the temporal characteristics of the states derived from the surrogate analysis were different compared to the real data.

Second, over half of the surrogate sets produced 2 modes of covariation when submitted to CCA. As phase randomization alters how the HMM fits each state's first- (i.e. activity) and second-order (i.e. FC) statistics, but keeps the static FC properties the same, it is likely that some modes of covariation in the surrogate data reflect information held by static FC properties that are in part likely to be additionally represented by patterns of time-varying FC as seen in the real data as well.

Third, the similarity of the 1st discovered mode was much greater than the similarity of the 2nd discovered mode between the surrogate data and real data. Given the similarity in the real data between the 1st time-varying mode and the single static FC mode, the 1st mode likely represents the relationship seen when using static FC data. Thus, time-varying FC is likely to hold information represented by *both* static FC as well as time-varying FC. As phase-randomization does not alter the static FC profile of the data, it is likely that the HMM was able to capture this information when fit to the phase-randomized surrogates in the same way as it was able to capture it in the real data.

Fourth, across both modes, the similarity of the behavior-respective post-hoc correlations between the surrogate and real data was much greater than the similarity of the time-varying FC-respective post-hoc correlations. The degree of dissimilarity for time-varying FC is likely a direct result of the phase-randomization procedure specifically altering crucial components of the time-varying FC architecture seen within each subject. However, the greater similarity of the behavioral profiles is potentially indicative that stable, and most likely static, FC components in the resting-state timeseries are what drive interindividual

behavioral relationships. With that said, the likely interaction between mode (i.e. 1 vs. 2) and data profile type (i.e. behavior- vs. time-varying FC-respective post-hoc correlations) still indicates that time-varying FC, as captured by our HMM, holds information that is unique and explanatory beyond that captured by static FC.

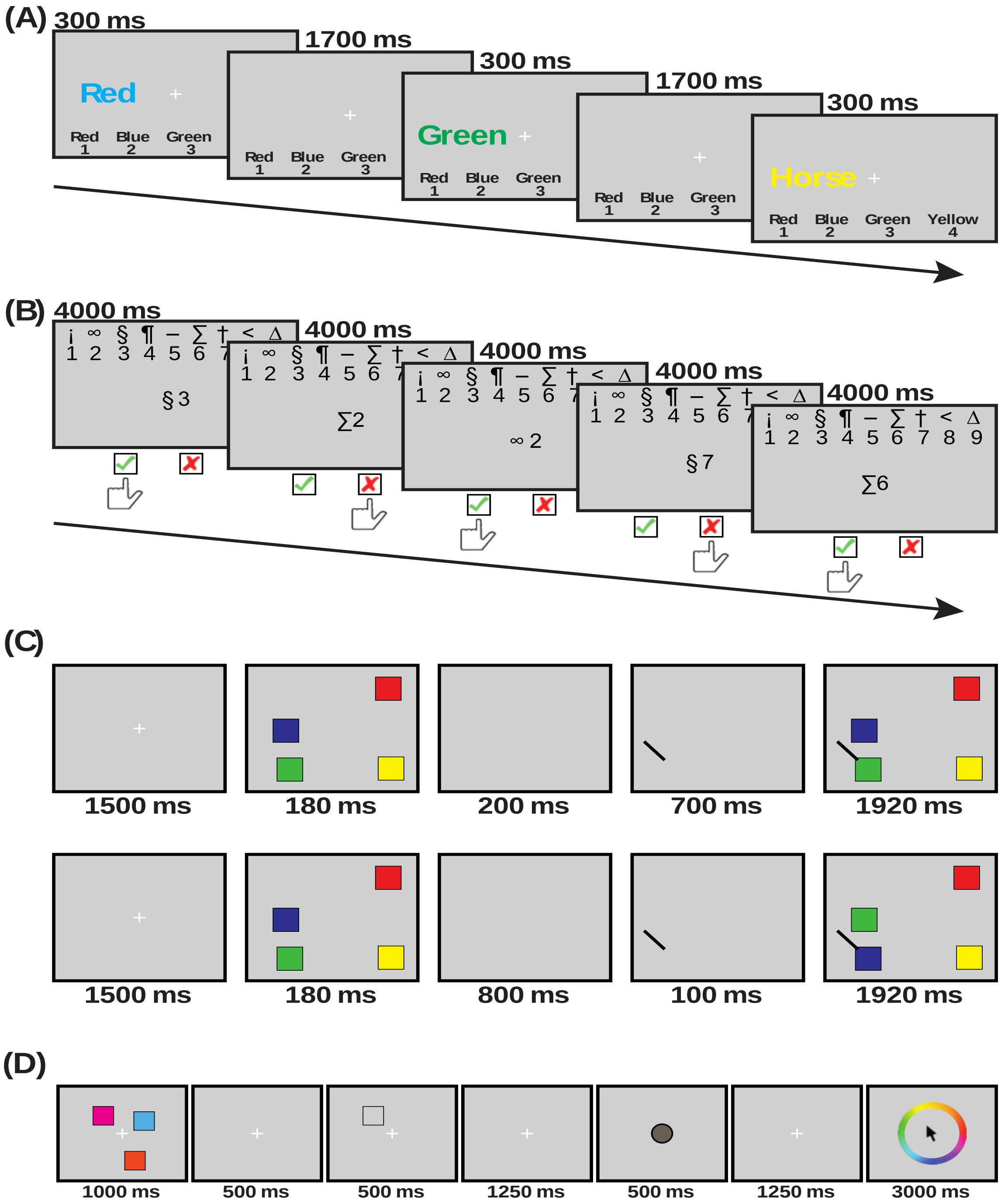

Figure S1. Computerized Task Paradigms. Shown are the time courses of example trials of the 4 computerized behavioral tasks. (A) Stroop task. Trial 1 is Incompatible, 2 is Compatible, and 3 is Neutral. (B) DSST. (C) Spatial WM. Both early- and late-cueing trial types are shown, with the early-cueing trial depicting a match trial and the late-cueing trial depicting a non-match trial. (D) Color WM.

	Processing RT	Task General	Working Mem. RT	Working Mem. Precision RT	Affect	Processing Accuracy	Arousal	Impulsivity
Spatial WM Med. RT (Match)	-0.07	-0.16	**0.81**	-0.18	0.04	0.14	0.03	0.03
Spatial WM % Acc. (Match)	0.20	**0.55**	0.38	0.03	-0.00	-0.20	-0.13	0.35
Spatial WM Std. RT (Match)	-0.04	0.18	**0.45**	0.12	0.01	0.12	0.05	0.09
Spatial WM Med. RT (Non-Match)	-0.09	-0.03	**0.86**	-0.10	-0.08	-0.12	-0.02	-0.11
Spatial WM % Acc. (Non-Match)	-0.10	-0.04	-0.21	-0.12	0.39	0.07	-0.10	-0.15
Spatial WM Std. RT (Non-Match)	-0.01	-0.01	**0.57**	0.27	0.21	-0.04	-0.09	-0.04
Stroop Med. RT	-0.06	**-0.84**	0.06	-0.12	-0.30	0.07	-0.16	0.02
Stroop % Acc.	-0.11	**0.65**	-0.18	-0.05	-0.05	-0.37	0.16	-0.07
Stroop Std. RT	0.13	0.21	-0.08	-0.21	0.10	0.24	-0.02	0.05
DSST Med. RT (Match)	**0.98**	-0.06	0.00	0.09	0.05	-0.16	-0.26	-0.03
DSST % Acc. (Match)	-0.10	**-0.61**	-0.03	-0.07	0.05	**1.16**	-0.01	0.07
DSST Std. RT (Match)	**0.58**	0.17	-0.02	-0.15	-0.08	-0.09	0.12	-0.12
DSST Med. RT (Non-Match)	**1.03**	0.00	-0.08	-0.05	0.17	0.05	-0.06	-0.10
DSST % Acc. (Non-Match)	0.11	**-0.55**	-0.18	0.14	-0.07	0.15	-0.17	0.24
DSST Std. RT (Non-Match)	**0.62**	0.04	-0.17	-0.17	-0.23	-0.10	0.15	0.11
Color WM Med. Error	0.15	0.16	0.15	-0.23	0.37	0.42	0.12	-0.07
Color WM Med. RT	-0.04	0.13	-0.08	**0.92**	0.05	-0.16	0.03	0.02
Color WM Std. Error	0.00	0.21	0.07	-0.13	0.10	0.18	0.01	-0.11
Color WM Std. RT	-0.02	-0.02	-0.06	**0.91**	0.20	0.05	0.03	-0.17
Anxious	-0.17	**0.56**	-0.14	0.04	0.43	-0.05	-0.16	0.09
Happy	-0.07	-0.15	-0.07	-0.17	**-0.68**	0.05	0.00	0.12
Sad	0.11	0.28	0.01	0.10	**0.85**	0.06	-0.00	-0.01
Nauseous	0.03	-0.09	-0.08	0.04	0.38	-0.43	0.03	-0.00
Drowsy	-0.24	0.13	-0.16	0.13	-0.07	0.10	**0.85**	0.07
Jittery	-0.33	0.29	-0.16	-0.30	0.19	-0.18	-0.20	0.25
Fatigued	-0.06	0.20	0.06	-0.03	0.02	-0.07	**0.98**	-0.08
Dizzy	0.08	-0.29	0.13	-0.39	0.31	-0.33	0.13	-0.04
BIS - Attention	0.16	0.03	-0.17	0.06	0.23	0.12	0.09	**0.46**
BIS - Motor	-0.13	-0.03	-0.04	-0.12	-0.11	0.06	-0.05	**1.05**
BIS - Non-Planning	0.07	-0.24	0.17	0.10	0.20	-0.05	0.31	**0.44**
Box Completion Time	0.24	0.03	-0.25	0.17	0.09	0.20	0.01	-0.04

Factor Loading (color scale): 1.0, 0.8, 0.6, 0.4, 0.2, 0, -0.2, -0.4, -0.6, -0.8, -1.0

Figure S2. Factor Loadings in Behavioral Data. Shown are the factor loadings across the 8 derived factors for each of the 31 behavioral measures. To aid interpretability, certain values are highlighted to show the clustering of loadings within groups. Factors were assigned names based on the behavioral variables that loaded most heavily on them. RT: Reaction Time.

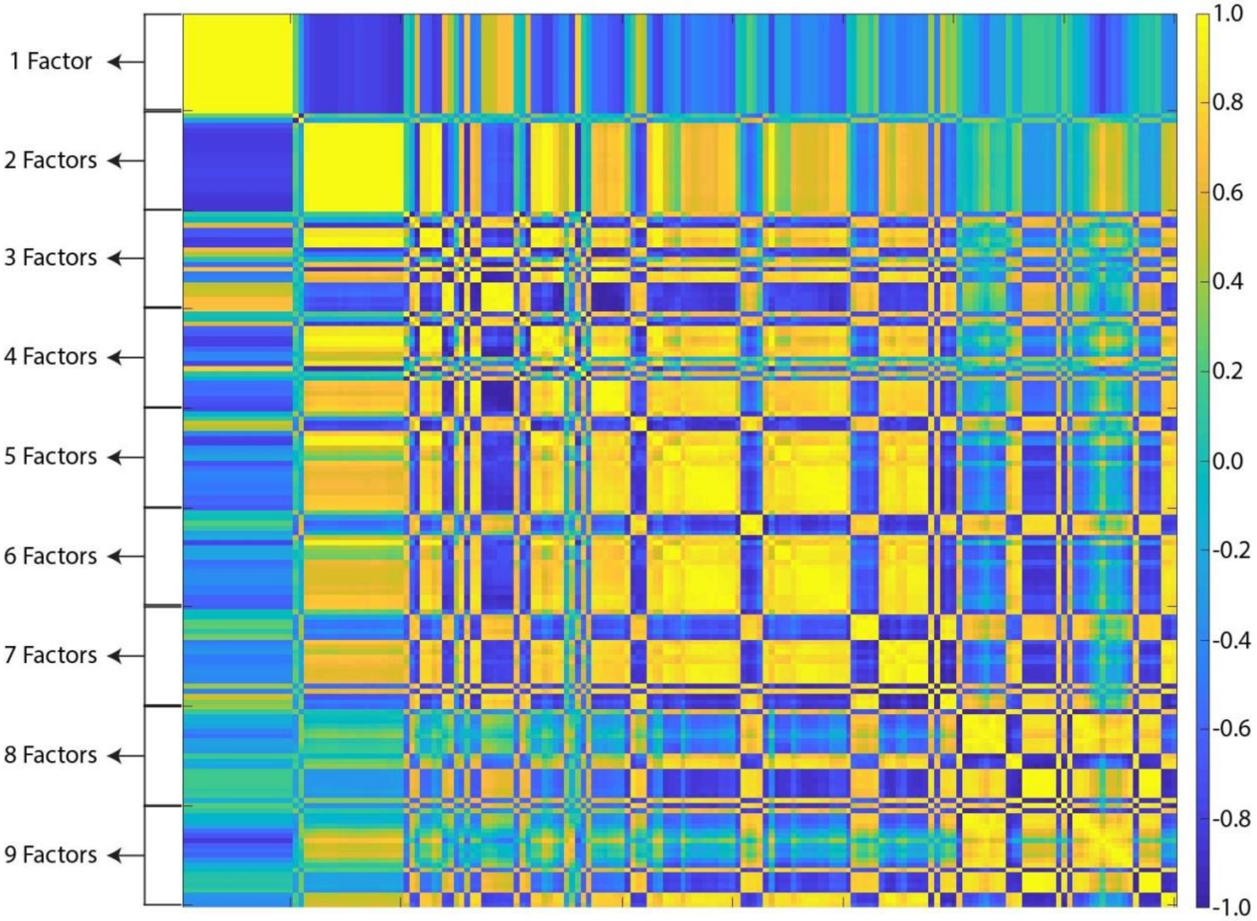

Figure S3. Validation of Static Functional Connectivity CCA. Similarity between modes of covariation are plotted across varying numbers of behavioral factors (1-9) and edge strength principle components (1-20). Similarity was measured as the Pearson correlation coefficient between each mode's vector of 31 behavioral post-hoc correlations. The plot is organized such that for each CCA run, connectivity data in varying principal component space is grouped together within chunks of behavioral data in a fixed factor-space. For example, the first row represents CCA being run on behavioral data in 1-factor space and connectivity data in 1-PC space; the 10th row represents CCA being run on behavioral data in 1-factor space and connectivity data in 10-PC space; the 25th row represents CCA being run on behavioral data in 2-factor space and connectivity data in 5-PC space, etc. Essentially, only one mode is found across all PCs when in 1- and 2-factor space, with the discovered modes being identical within factor space, and almost exact inverses of each other across factor spaces. Modes discovered from CCA run on data in 3-factor space and above, across all PCs, almost entirely represent the same relationship as each other.

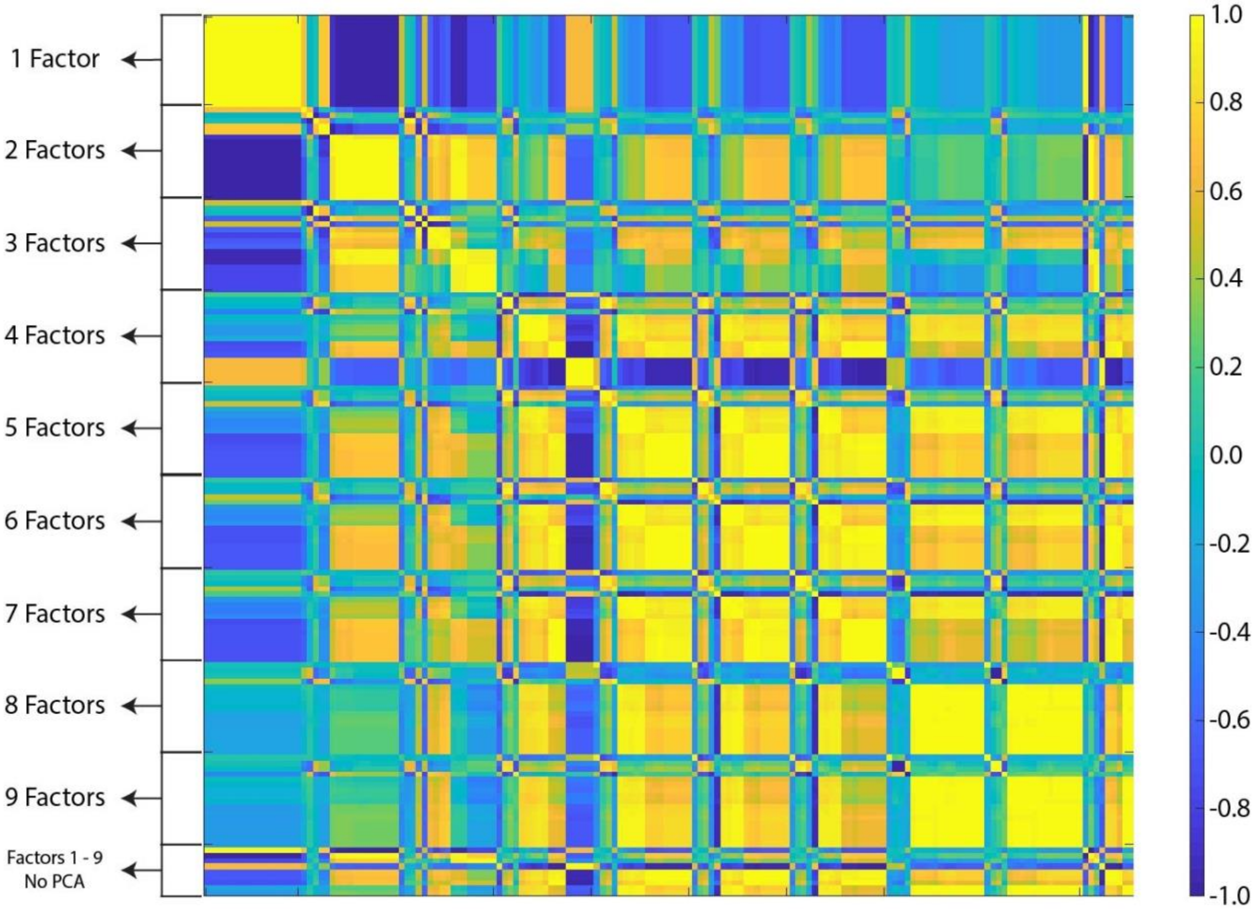

Figure S4. Validation of Time-varying Functional Connectivity CCA. Similarity between modes of covariation are plotted across varying numbers of behavioral factors (1-9) and edge strength principle components (1-17, capped by number of HMM variables included). Similarity was measured in the same way as for the static FC CCA validation procedure using Pearson correlation. Unlike in the static FC case, we additionally assessed whether the discovered modes differed as a function of whether PCA was, or was not, run on the HMM data. The final 9 rows represent the modes discovered from CCA run on behavioral data in 1- through 9-factor space when PCA was not performed on the HMM data. As can be seen, the modes discovered when CCA was run on the raw HHM data almost perfectly match those found when PCA was run on the HMM data. A corresponding plot for post-hoc correlations computed from modes greater than 1 is inappropriate as we had no *a priori* knowledge that more than 1 significant mode of covariation would be discovered, and thus only the first mode of covariation can be compared across datasets.

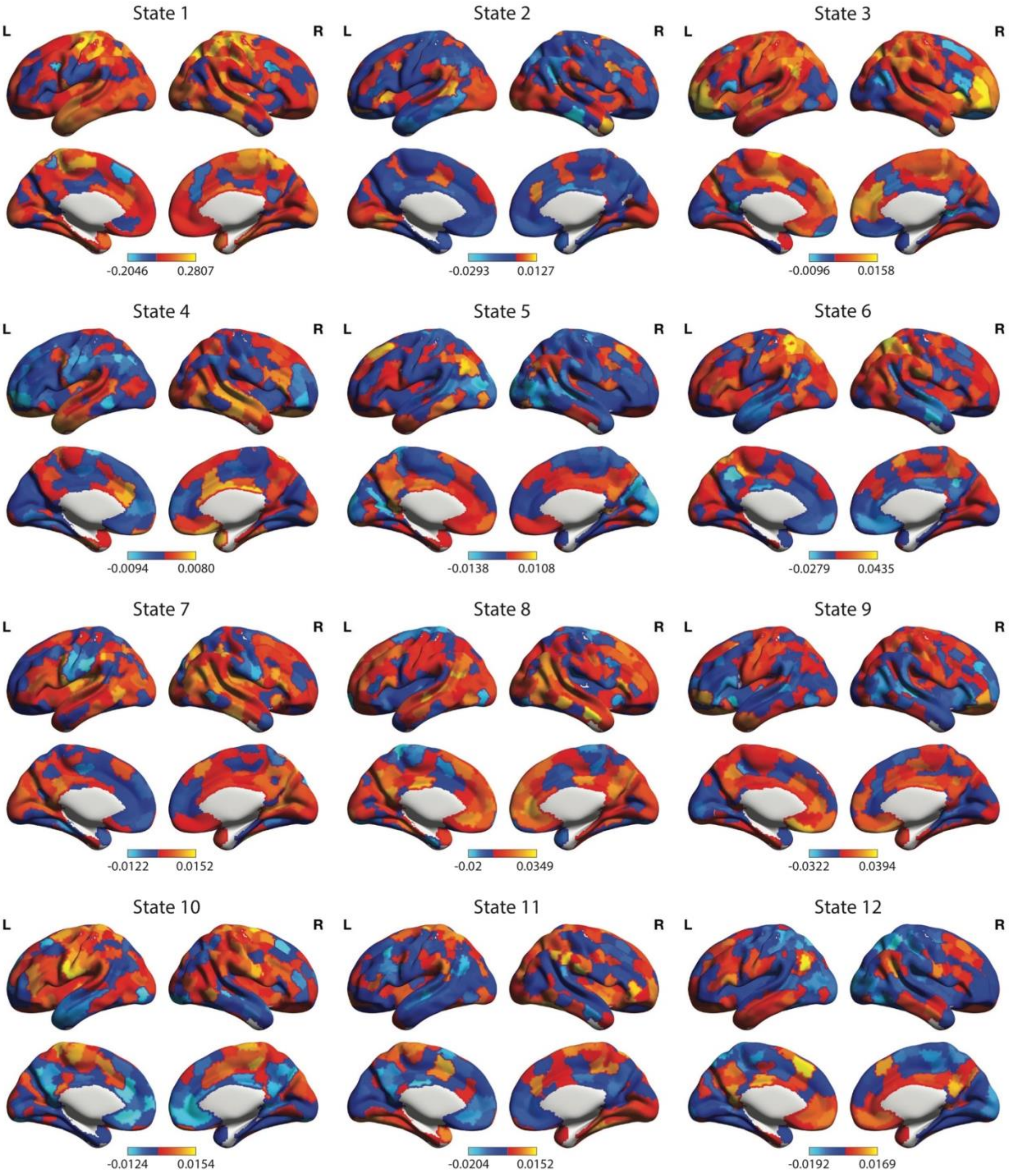

Figure S5. Spatial profile of HMM States. Shown are the mean activation profiles (positive and negative) for each of the 12 HMM states. States 1 and 2 formed one metastate, states 3-11 formed another metastate, and state 12 clustered by itself.
